# Prognostic value of lymph node metastases of differentiated thyroid cancer (DTC) according to the local advancement and range of surgical excision

**DOI:** 10.1186/1756-6614-3-8

**Published:** 2010-10-29

**Authors:** Agnieszka Czarniecka, Michal Jarzab, Jolanta Krajewska, Ewa Chmielik, Bogna Szcześniak-Klusek, Ewa Stobiecka, Robert Kokot, Aleksander Sacher, Stanisław Poltorak, Jan Wloch

**Affiliations:** 1 M. Sklodowska-Curie Memorial Cancer Center and Institute of Oncology, Gliwice Branch, Gliwice, Poland

## Abstract

In differentiated thyroid carcinoma (DTC) with primary tumor smaller than 1 cm, the routine central lymph node (LN) dissection is questioned, due to increased risk of post-surgery complications and lack of confirmed benefit.

**Aim:**

The analysis of prognostic significance of LN metastases, in DTC patients to verify the potential role of central neck lymphadenectomy on disease staging.

**Materials and methods:**

The group of 195 DTC patients, primarily operated between 2004 and 2005, was retrospectively analyzed. 184 patients after radical operation, with no distant metastases diagnosed before surgery, were included into analysis. LN metastases were observed in 55 of cases (28%). In 124 cases only dissection of central LN compartment was performed, in 36 patients also uni- or bilateral modified cervical lymphadectomy was carried out. In 24 patients with tumor limited to the thyroid gland without suspicious lymph nodes, the routine central lymph node dissection was not done.

**Results:**

Median follow-up was 4 years. The 5-year overall and disease free survival standardized ratio were 100% and 95% respectively. The risk of LN metastases increased with the more locally advanced cancer. In the group of 124 patients, in whom only central LN dissection was performed, LN metastases were diagnosed in 15 cases (12%). No significant relation between multifocality and frequency of central and/or lateral LN metastases was noticed. Significant correlation between N feature and extrathyroidal invasion was observed (p = 0,0003). The presence of LN metastases was related to worsening of disease free survival from 99 to 90%. During the follow-up recurrence occurred in 6 (3%) cases. In 24 patients in whom only total thyroidectomy was done, no local or distant recurrence was observed. The assessment of early postoperative complications (hypoparathyroidism, paresis of vocal cords) indicated that the frequency of early calcium balance disturbances was significantly lower in patients in whom central LN dissection was not performed (p = 0,04)

**Conclusions:**

Our result indicate that in the early diagnosis of thyroid cancer, the occurrence of LN DTC metastases is rarer and was observed only in 12% of elective dissections of central LN node compartment, if no lateral dissection was indicated due to the lack of clinical suspicion. In DTC patients with tumor diameter <1 cm and no sonographical or inraoperative suspicion on LN involvement, routine central lymphadenectomy may be not obligatory.

## Introduction

The optimal extent of surgical approach related to cervical lymph nodes in DTC is still discussed. Up to now there is no agreement whether cervical lymph node metastases (N feature) are related to a worse prognosis in DTC patients, when only central lymph nodes or unilateral lateral lymph nodes are involved and the extent of lymphadenectomy is sufficient. Some data confirm the importance of this prognostic factor, its influence both on overall and disease free survival [1-5]. However, some data demonstrate its implication related to cancer relapse only [6-8] whereas other neglect its prognostic value [9-11]. Obviously, the issue is more important in papillary thyroid cancer (PTC) where lymph node metastases occur more frequently than in follicular thyroid cancer (FTC). Papers published in the recent years more and more frequently recommend the limited extent of central compartment lymph node dissection in very low risk DTC patients due to a higher risk of postsurgical complications as well as to the lack of confirmed benefit [12-17]. Thus, it seems that, to plan an optimal, stage-dependent extent of surgery, the significance of this factor should be taken into consideration.

The actual guidelines concerning DTC treatment recommend total thyroidectomy together with central compartment (group VI) dissection in almost all DTC patients. Lateral neck lymphadenectomy (group II - V) is indicated only if the presence of lymph node metastases is proven by preoperative FNA or by open biopsy during the surgery or, at least, clinically suspected. In the recent years there are more and more data suggesting the possibility of safe resignation of the routine central compartment dissection in low risk DTC [12-17]. The rationale for this is related to higher risk of postoperative complications depending on the extent of surgery (recent studies revealed higher ratio of early complications than it was previously observed). Simultaneously, no higher risk of relapse or cancer-related death was observed [12-17].

### The aim of the study

A retrospective analysis of the prognostic impact of lymph node metastases, particularly of the central neck compartment, in DTC patients.

## Materials and methods

The group of 195 DTC patients, primarily operated between 2004 and 2005 in Oncological Surgery Clinic in Gliwice, were retrospectively analyzed. In 185 radical operation (R0 resection) was carried out whereas in 10 non-radical approach (R2 resection) was performed due to a locally advanced disease. Distant metastases were diagnosed in 10 (5%) patients, among them in 7 (4%) preoperatively.

184 DTC patients who received radical operation, with no distant metastases diagnosed before surgery, were included into further, more detailed analysis. One subject with poorly differentiated thyroid cancer was excluded. The study group consisted of 160 (87%) women and 24 (13%) men, mean age 46 years (median 46 yrs). Primary total thyroidectomy was carried out in all patients. Among them there were 178 PTC (97%) and 6 (3%) FTC cases. 115 patients were in pT1 stage (63%), 29 in pT2 (16%), 34 in pT3 (18%) and 6 in pT4 (3%) (according to the UICC classification 2002). The extent of lymph node dissection is presented in Table 1. Lymph node metastases were observed in 51 (28%) (Table 2). Complementary radioiodine therapy was indicated in all patients routinely.

**Table 1:** Extent of lymph nodes operations.

Extent of lymph node operations	Number of patients	Frequency
No lymph node dissection	24	13%

Only central lymph node dissection (group VI)	124	67%

Central + unilateral cervical dissection	32	18%

Central + bilateral cervical dissection	4	2%

**Table 2:** The frequency of lymph node (N) and distant (M) metastases in relation to T stage.

T stage	Number of patients	N1	Frequency	M1	Frequency
T1	115	22	19%	0	0%

T2	29	8	27%	1	3%

T3	34	18	53%	3	9%

T4	6	3	50%	0	0%

**Total**	**184**	**51**	**28%**	**4**	**2%**

Mean and median follow-up was 4 yrs.

Statistical analysis was carried out by the use of SPSS 12 software. The chi^2 ^test and Kaplan-Meyer survival analysis were performed.

## Results

**Figure 1:**
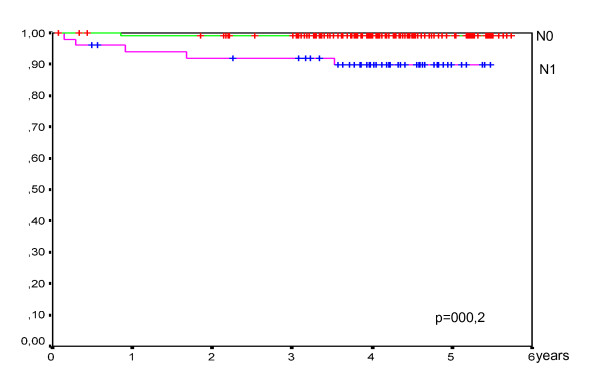
**Analysis of 5 years disease-free survival in relation to presence of neck lymph node metastases**.

The 5-year overall and disease free survival standardized ratio were 100% and 95% respectively. The presence of lymph node metastases was related to worsening of disease free survival from 99 to 90% and the difference was statistically significant (p = 0,002, Figure 1).

The risk of lymph node metastases increased with the more locally advanced cancer. The lowest ratio of lymph node involvement, 19%, was observed in pT1 DTC patients (tumor diameter ≤2 cm) (Table 2). Definitely, lymph node metastases coexisted most commonly with extracapsular invasion in pT3 and pT4 (55%) tumors. Significant correlation between N feature and extrathyroidal invasion was observed (p = 0,0003). Multifocal cancer growth was present in 47 (26%) subjects. However, no significant relation between multifocality and frequency of lymph node metastases was noticed.

During the follow-up cancer recurrence occurred in 6 (3%) subjects, 5 of them were from N1 subgroup. Local relapse in 2 of them, whereas in 4 distant metastases were diagnosed.

In the subgroup of 124 patients, in whom only central lymph node dissection was performed, lymph node metastases were diagnosed in 15 cases (12%). There were 6 pT1 subjects, 2 pT2 and 7 pT3 in this group. The tumor diameter smaller than 1 cm was not observed in any pT1 patient, in 1 patient the tumor diameter was 1 cm, in another one 1.2 cm and in 4 patients 2 cm. In the subgroup of 124 patients who received thyroidectomy and central lymphadenectomy, 2 cancer recurrences occurred, one local relapse in pT3N1 patient and 1 case of bone metastases revealed in whole body scintigraphy after therapeutic dose of 131-I in pT2N0 patient

In 24 patients with tumor limited to the thyroid (pT1 and pT2), with no suspicious lymph nodes during the surgery, the routine central lymph node dissection was not done. No recurrence was observed in this group.

The assessment of early postoperative complications (hypoparathyroidism as well as paralysis or paresis of vocal cords) during the first or the second day after the surgery was also carried out. The frequency of early calcium balance disturbances was significantly lower in patients in whom lymph node dissection was not performed (p = 0,04; Table 3.).

**Table 3:** Frequency of early postoperative complications observed in the first or second day after surgery.

	All patients (184)	Only central lymph node dissection (124)	Only total thyroidectomy (24)	p
**Paralysis or paresis of vocal cord**	20 (11%)	10 (10%)	0 (0%)	n.s.

**Hypoparathyroidism**	37 (20%)	29 (23%)	1 (4%)	p = 0,04

## Discussion

The importance of lymph node metastases in DTC has been discussed for many years. Our previous analysis, carried out on numerous, representative for Polish population group of 1141 DTC patients, showed that the presence of lymph node metastases affected significantly both disease free and overall survival during 10-year follow-up (p < 0,0001) [5]. Multivariate backward stepwise regression analysis demonstrated that lymph node metastases, against a background of other independent factors, were related to an increase of the risk of both cancer-related death and cancer relapse. In patients with lymph node involvement the risk of cancer-related death grew more than 3 times (RR 3,16). The risk of cancer recurrence was even higher - RR 4,48. Prospective randomized studies are lacking due to DTC relatively long-term natural course. Thus, present observational study is based on a retrospective analysis of patients treated and followed up in a uniform way in one center.

The influence of lymph node metastases on disease free survival during the 5-year follow-up of DTC patients was analyzed. The frequency of lymph node metastases was correlated with the tumor diameter (T feature). Lymph node involvement was observed more often in patients with extrathyroid tumor invasion (pT3 and pT4). Thus, resignation from central lymph node dissection seems to be safe only in low advanced PTC. However, central lymph node metastases were found in 6 pT1 subjects in the group of 15 patients in whom routine central neck lymphadenectomy revealed the presence of central lymph node metastases. However, in none of them the tumor diameter was <1 cm. Thus, at the beginning, it seems reasonable to restrict the extent of the operation only in patients with papillary microcarcinoma (intrathyroid tumor ≤1 cm) without clinically suspicion of central lymph node involvement. However, the cases of both central and lateral lymph node metastases in DTC patients with tumor <1 cm, particularly in children and young adults have to be not missed. Thus, a careful pre- and intraoperative evaluation of the suspected lymph nodes is necessary, associated with a detailed analysis of all clinical factors. This strategy has been adopted in our center and among 39 tumors of <1 cm diameter diagnosed preoperatively central lymphadenectomy was decided in 15 cases and was positive in none of them.

In no subject from the pilot group of 24 DTC patients, in whom routine central neck dissection was not done, cancer recurrence was diagnosed. On the other hand, clearly lower incidence of early postoperative complications was stated in this group, statistically significant as far as calcium-phosphorous balance is concerned [Table 3]. Similar observation was reported by Palestini at al. In the group of 64 patients after total thyroidectomy with bilateral central lymphadenectomy the laryngeal recurrent nerve paresis was present in 5 (7,8%) subjects, whereas hypoparathyroidism in 20 (31%). In the group of 93 subjects after total thyroidectomy with unilateral central lymphadenectomy (on the tumor side) these complications concerned 5 (5,4%) and 25 (27%) patients, respectively. The lowest complication ratio was observed in the group of 148 patients in whom only total thyroid resection was carried out - laryngeal recurrent nerve paresis in 13 (9%) and hypoparathyroidism in 19 (13%) cases. The authors reported statistically significant difference in frequency of calcium-phosphorus balance disturbances depending on the extent of the surgery (p = 0,003) [17].

Due to the number of patients and follow-up period our study should be considered as an initial report. However, it seems that after proper selection in some cases routine central lymph node dissection may and should be avoided. According to the Italian authors aiming restriction of the parathyroid vascular shock in justified cases unilateral central lymphadenectomies (on the tumor side) may be performed, even in more advanced stages [17].

Probably, the cancer expansion ability (lymph node and distant metastasis) is related not only to its local advancement or tumor diameter but also to its molecular profile. Perhaps, the future evaluation of DTC molecular profile and its metastasizing ability may lead to a better planning of the treatment approach [18]. Unfortunately, up to now this decision is based on clinical and pathological features only.

## Conclusions

Our result indicate that in the last years, with the early diagnosis of thyroid cancer, the occurrence of lymph node DTC metastases is rarer and was observed only in 12% of elective dissections of central neck lymph node compartment, if no lateral dissection was indicated due to the lack of clinical suspicion. In DTC patients with tumor diameter <1 cm and no sonographical or inraoperative suspicion on lymph node involvement, routine central lymphadenectomy may be not obligatory.

## Competing interests

The authors declare that they have no competing interests.

## Authors' contributions

AC created the design of the study, performed surgical treatment, worked out the study material, performed the statistical analysis and drafted the manuscript. MJ created the design of the study, performed the statistical analysis. JK worked out the study material, drafted the manuscript. EC carried out histopathological assessment. BS-K carried out histopathological assessment. ES carried out histopathological assessment. RK worked out the study material. AS performed surgical treatment. SP performed surgical treatment. JW performed surgical treatment. All authors read and approved the final manuscript.
